# Bacteria-Stimulated Metamorphosis: an Ocean of Insights from Investigating a Transient Host-Microbe Interaction

**DOI:** 10.1128/msystems.00754-21

**Published:** 2021-08-31

**Authors:** Nicholas J. Shikuma

**Affiliations:** a Department of Biology and Viral Information Institute, San Diego State University, San Diego, California, USA

**Keywords:** contractile injection systems, MACs, host-microbe interaction, larvae, marine microbiology, metamorphosis, natural products, symbiosis

## Abstract

Recent research on host-microbe interactions has focused on intimate symbioses. Yet transient interactions, such as the stimulation of animal metamorphosis by bacteria, can have significant impacts on each partner. During these short-lived interactions, swimming animal larvae identify a desirable location on the seafloor and undergo metamorphosis into a juvenile based on the presence of specific bottom-dwelling bacteria. While this phenomenon is critical for seeding new animals to establish or maintain benthic ecosystems, there is an ocean of fundamental questions that remain unanswered. Here, I propose an updated model of how bacteria stimulate animal metamorphosis based on evidence that bacteria inject a stimulatory protein that prompts tubeworm metamorphosis. I consider what we hope to learn about stimulatory bacterial products, how animals recognize these products, and the consequences for both partners. Finally, I provide examples of how studying an enigmatic host-microbe interaction can serve as an engine for scientific discovery.

## BACTERIA-STIMULATED METAMORPHOSIS: AN ENIGMATIC HOST-MICROBE INTERACTION

**FIG 1:**
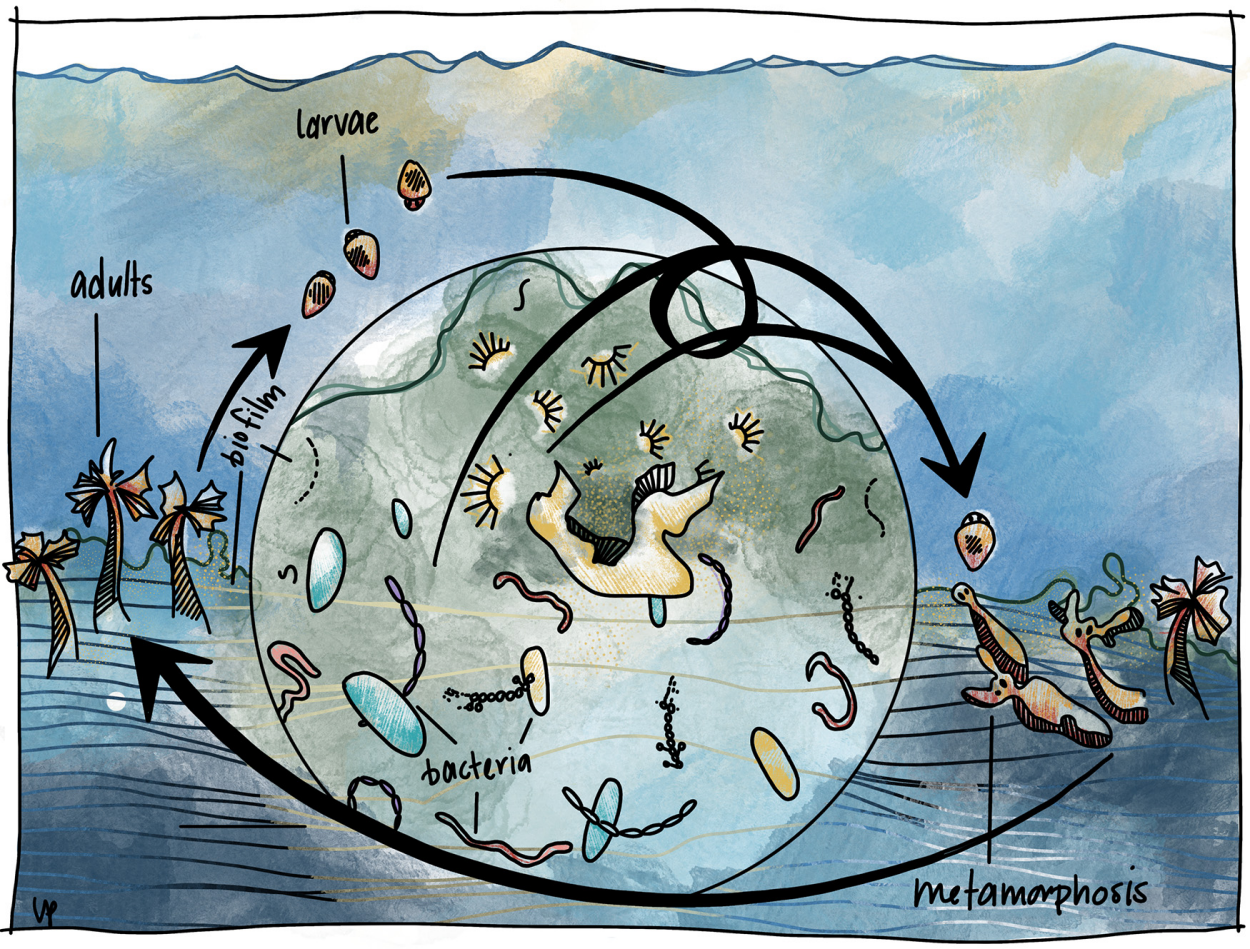
Illustration of the stimulation of animal metamorphosis by bacteria. The swimming larvae of bottom-dwelling marine animals (labeled larvae), such as tubeworms, identify a suitable location on the seafloor to live out their juvenile and adult life (labeled adults) based in part on the presence of specific bacteria embedded within a surface-bound biofilm. Some bacteria within stimulatory biofilms create products that promote the settlement and metamorphosis (labeled metamorphosis) of marine larvae. Bacteria and their stimulatory products are depicted within the central zoomed-in circle, and their location on the substratum is indicated by the biofilm label. Figure illustration and design by Leah Pantéa (Wholon; printed with permission under Creative Commons license).

Recent research on host-microbe interactions has focused on intimate symbioses, where partners in close contact can promote both pathogenic and beneficial outcomes. However, it has become clear that environmental bacteria can also provide cues during transient interactions that regulate essential processes in diverse animals (1). These fleeting host-microbe interactions have shaped animal development in an array of animal and microbial lineages; however, the mechanisms that underpin these interactions remain mysterious. A currently understudied example of one such transient microbe-animal interaction is the stimulation of animal metamorphosis by bacteria (2). During these interactions in marine environments, swimming animal larvae identify a suitable location on the seafloor and undergo metamorphosis into a juvenile based in part on the presence of specific bottom-dwelling bacteria forming biofilms attached to submerged surfaces (Fig. 1). Representative animals from each major branch of the animal tree of life have been shown to undergo metamorphosis in response to bacteria (e.g., corals, tubeworms, and urchins) (3–5). It is therefore plausible that the phenomenon of bacteria-stimulated metamorphosis evolved long ago and continues to shape where and when marine animal larvae undergo metamorphosis.

In marine habitats, bacteria-stimulated metamorphosis is a critical process for seeding new animals to maintain or establish populations. This process might, in part, dictate the distribution of animals and ecosystems in the environment. Bacteria-stimulated metamorphosis likely contributes to the economically costly process of biofouling of ship hulls (6) and is important for the life cycle of aquaculture species such as oysters (7). This phenomenon is not restricted to marine animals, as symbiotic bacteria have also been implicated in insect metamorphosis (8), and an analogous phenomenon occurs when seaweed zoospores settle in response to bacteria (9). Despite the importance of this process, three major questions about the phenomenon remain unanswered: (i) What are the bacterial products that promote metamorphosis? (ii) How do animals recognize bacterial products? (iii) Which partners benefit from this interaction and how (i.e., commensalism, mutualism, parasitism)? To address these questions, my research program uses emerging host-microbe model interactions to determine the mechanistic basis of bacteria-stimulated metamorphosis.

## A SURPRISINGLY DIFFERENT WAY THAT BACTERIA STIMULATE METAMORPHOSIS

Historically, bacterial products that stimulate metamorphosis were described as small soluble molecules or products associated with the bacterial cell surface or biofilm matrices (10, 11). However, we discovered a surprisingly different way that some bacteria stimulate animal metamorphosis; the bacterium Pseudoalteromonas luteoviolacea produces syringe-like structures called metamorphosis-associated contractile structures (MACs) that inject stimulatory proteins into target animals (12). MACs belong to a class of syringe-like structures termed contractile injection systems (CIS) that bear homology to the contractile tails of bacteriophage (the viruses of bacteria) and are produced by diverse bacteria (13, 14). CIS often translocate protein payloads into target cells, which may target a specific molecule or process to exert their effect. Until our recent work, CIS were known to mediate antagonism among competing microbes or between bacteria and their eukaryotic hosts (15). However, we discovered that a single protein loaded within the MACs complex is translocated into the larvae of a tubeworm, Hydroides elegans, and stimulates normal metamorphosis (16, 17). This single protein effector, termed metamorphosis-inducing factor 1 (Mif1), represents the first protein from a bacterium identified to stimulate the metamorphosis of an animal.

## AN OCEAN OF POSSIBILITIES

Bacteria from diverse lineages have been shown to stimulate the metamorphosis of marine animal larvae. These bacteria include strains from the *Gammaproteobacteria* and *Alphaproteobacteria* classes, as well as bacteria from the *Bacteroidetes* group and Gram-positive *Firmicutes* phylum (5, 18). Intriguingly, some bacteria are potent stimulants of metamorphosis, while closely related strains can be unable to stimulate metamorphosis under equivalent conditions. The diversity of bacterial species that stimulate metamorphosis suggests that there are numerous stimulatory bacterial products remaining to be discovered.

So far, genes for the biosynthesis of two products from bacteria that stimulate metamorphosis have been identified. These genes promote the biosynthesis of a brominated natural product tetrabromopyrrole (TBP) or encode Mif1, which stimulate coral and tubeworm metamorphosis, respectively (10, 16). Some bacteria possess the genes and ability to produce both TBP and Mif1 (19). These products are chemically different; TBP is a brominated aromatic hydrocarbon, while Mif1 is a proteinaceous effector. Yet both have been shown to stimulate metamorphosis, suggesting that very different bacterial products can promote a dramatic developmental transition in diverse animals.

A small number of purified products from bacteria have been shown to stimulate the metamorphosis of the tubeworm, Hydroides elegans, and the cnidarian, Hydractinia echinata. These products include outer membrane vesicles, lipopolysaccharides (LPS), extracellular polysaccharides, and lysophospholipids (11, 20, 21). These products induce metamorphosis when provided to larvae in a purified form, but it is currently unknown if the products stimulate metamorphosis when produced by, and in the context of, whole bacteria. Whether animal larvae respond to specific bacterial products from living bacteria within biofilms will be an interesting avenue of future research.

## AN UPDATED MODEL OF BACTERIA-STIMULATED METAMORPHOSIS

The phenomenon that bacteria stimulate animal metamorphosis was discovered over 80 years ago (22). Since this initial discovery, one model explaining how bacteria stimulate metamorphosis has gained traction (2). This model predicts that animals are stimulated to undergo metamorphosis in response to bacterial products that result from normal growth or metabolism. This model implies an “animal-driven” process where bacteria serve as passive features of the environment that animals use as an indicator of a preferable habitat. It is unknown whether this animal-driven model explains most of the interactions mediating bacteria-stimulated metamorphosis in the environment. Our discovery that P. luteoviolacea stimulates tubeworm metamorphosis by producing a CIS that translocates a bioactive protein into the animal larvae builds on the previous model of how bacteria stimulate metamorphosis. This bioactive protein mechanism implies that a “bacteria-driven” process also exists where bacteria drive the interaction by injecting stimulatory proteins into animal larvae. Importantly, both mechanisms of bacteria-stimulated metamorphosis might exist simultaneously in the environment and drive the process of animal recruitment to new habitats. This updated model of how bacteria stimulate animal metamorphosis leads to broader questions of whether and how the partners of this transient interaction are harmed or benefit from the encounter (23). Whether biofilm bacteria that stimulate metamorphosis colonize the juvenile and adult organism was questioned only recently (24) and remains a critical gap in knowledge about the relationship.

## ANTICIPATED ADVANCES IN THE NEXT 5 YEARS AND BEYOND

Within the next 5 years, I envision that there will be three main advances that will push the field of studying bacteria-stimulated metamorphosis forward as follows: (i) Diverse bacterial products that stimulate animal metamorphosis and the genetic basis of their biosynthesis will be identified. These products will differ in their chemical composition, structure, and function (e.g. a specialized metabolite such as tetrabromopyrrole or a protein effector, like Mif1). The mode of action of the bacterial product and the mode of perception by the larvae will vary widely. (ii) Model systems focused on microbe-animal symbioses will be used more frequently, and methods to study them will catch up to established model organisms. Technical advances to study the animal partners will help to determine how animal larvae perceive bacterial products that stimulate metamorphosis. (iii) Discoveries about the diverse bacterial products that stimulate metamorphosis and how animals recognize these products will help to flesh out the broader question of whether the relationship between the bacteria and animal partners is a parasitic, commensal, or mutualistic interaction. It remains unknown whether bacteria that stimulate metamorphosis continue their interaction with the animal once it has undergone metamorphosis.

## UNEXPECTED INSIGHTS FROM STUDYING BACTERIA-STIMULATED METAMORPHOSIS

Studying a mysterious host-microbe phenomenon has led to two unexpected insights. First, in addition to targeting tubeworm larvae, we found that MACs are capable of targeting very different types of eukaryotic cells, including insect cells and mouse macrophages, *ex vivo* (25). CIS such as MACs might therefore be amenable to engineering for biotechnology purposes as protein delivery devices to target eukaryotic cells. Second, while studying CIS that promote tubeworm metamorphosis, my lab made a fortuitous discovery—we found that a poorly described class of CIS genes is present within *Bacteroidales* bacteria from the gut microbiomes of nearly all healthy human adults from the United States and Europe (14). Further, we show that individuals suffering from irritable bowel disease have fewer CIS genes than healthy individuals, hinting at their role in human health. Our discoveries provide important instances of the power of fundamental research as an engine for scientific discovery.

## CONCLUSION

Numerous marine bacteria forming multispecies biofilm communities on submerged surfaces likely serve as an indicator of a preferable habitat for, and trigger the metamorphosis of, marine larvae. Studying such interactions will provide a wealth of foundational knowledge with profound health, economic, and biotechnology applications.

## References

[1] Woznica A, King N. 2018. Lessons from simple marine models on the bacterial regulation of eukaryotic development. Curr Opin Microbiol 43:108–116. doi:10.1016/j.mib.2017.12.013. 29331767 PMC6051772

[2] Cavalcanti GS, Alker AT, Delherbe N, Malter KE, Shikuma NJ. 2020. The influence of bacteria on animal metamorphosis. Annu Rev Microbiol 74:137–158. doi:10.1146/annurev-micro-011320-012753. 32905754

[3] Huggett MJ, Williamson JE, de Nys R, Kjelleberg S, Steinberg PD. 2006. Larval settlement of the common Australian sea urchin *Heliocidaris erythrogramma* in response to bacteria from the surface of coralline algae. Oecologia 149:604–619. doi:10.1007/s00442-006-0470-8. 16794830

[4] Sneed JM, Sharp KH, Ritchie KB, Paul VJ. 2014. The chemical cue tetrabromopyrrole from a biofilm bacterium induces settlement of multiple Caribbean corals. Proc Biol Sci 281:20133086. doi:10.1098/rspb.2013.3086. 24850918 PMC4046396

[5] Unabia CRC, Hadfield MG. 1999. Role of bacteria in larval settlement and metamorphosis of the polychaete *Hydroides elegans*. Mar Biol 133:55–64. doi:10.1007/s002270050442.

[6] Dobretsov S, Abed RMM, Teplitski M. 2013. Mini-review: inhibition of biofouling by marine microorganisms. Biofouling Routledge 29:423–441. doi:10.1080/08927014.2013.776042. 23574279

[7] Yu X, He W, Li H, Yan Y, Lin C. 2010. Larval settlement and metamorphosis of the pearl oyster *Pinctada fucata* in response to biofilms. Aquaculture 306:334–337. doi:10.1016/j.aquaculture.2010.06.003.

[8] Hammer TJ, Moran NA. 2019. Links between metamorphosis and symbiosis in holometabolous insects. Philos Trans R Soc Lond B Biol Sci 374:20190068. doi:10.1098/rstb.2019.0068. 31438811 PMC6711286

[9] Singh RP, Reddy CRK. 2014. Seaweed-microbial interactions: key functions of seaweed-associated bacteria. FEMS Microbiol Ecol 88:213–230. doi:10.1111/1574-6941.12297. 24512602

[10] El Gamal A, Agarwal V, Diethelm S, Rahman I, Schorn MA, Sneed JM, Louie GV, Whalen KE, Mincer TJ, Noel JP, Paul VJ, Moore BS, El Gamal A, Agarwal V, Diethelm S, Rahman I, Schorn MA, Sneed JM, Louie GV, Whalen KE, Mincer TJ, Noel JP, Paul VJ, Moore BS. 2016. Biosynthesis of coral settlement cue tetrabromopyrrole in marine bacteria by a uniquely adapted brominase-thioesterase enzyme pair. Proc Natl Acad Sci USA 113:3797–3802. doi:10.1073/pnas.1519695113. 27001835 PMC4833250

[11] Freckelton ML, Nedved BT, Hadfield MG. 2017. Induction of invertebrate larval settlement; different bacteria, different mechanisms? Sci Rep 7:42557. doi:10.1038/srep42557. 28195220 PMC5307369

[12] Shikuma NJ, Pilhofer M, Weiss GL, Hadfield MG, Jensen GJ, Newman DK. 2014. Marine tubeworm metamorphosis induced by arrays of bacterial phage tail-like structures. Science 343:529–533. doi:10.1126/science.1246794. 24407482 PMC4949041

[13] Chen L, Song N, Liu B, Zhang N, Alikhan NF, Zhou Z, Zhou Y, Zhou S, Zheng D, Chen M, Hapeshi A, Healey J, Waterfield NR, Yang J, Yang G. 2019. Genome-wide identification and characterization of a superfamily of bacterial extracellular contractile injection systems. Cell Rep 29:511–521.e2. doi:10.1016/j.celrep.2019.08.096. 31597107 PMC6899500

[14] Rojas MI, Cavalcanti GS, McNair K, Benler S, Alker AT, Cobián-Güemes AG, Giluso M, Levi K, Rohwer F, Bailey BA, Beyhan S, Edwards RA, Shikuma NJ. 2020. A distinct contractile injection system gene cluster found in a majority of healthy adult human microbiomes. mSystems 5:e00648-20. doi:10.1128/mSystems.00648-20. 32723799 PMC7394362

[15] Ho BT, Dong TG, Mekalanos JJ. 2014. A view to a kill: the bacterial type VI secretion system. Cell Host Microbe 15:9–21. doi:10.1016/j.chom.2013.11.008. 24332978 PMC3936019

[16] Ericson CF, Eisenstein F, Medeiros JM, Malter KE, Cavalcanti GS, Zeller RW, Newman DK, Pilhofer M, Shikuma NJ. 2019. A contractile injection system stimulates tubeworm metamorphosis by translocating a proteinaceous effector. Elife 8:e46845. doi:10.7554/eLife.46845. 31526475 PMC6748791

[17] Shikuma NJ, Antoshechkin I, Medeiros JM, Pilhofer M, Newman DK. 2016. Stepwise metamorphosis of the tubeworm *Hydroides elegans* is mediated by a bacterial inducer and MAPK signaling. Proc Natl Acad Sci USA 113:10097–10102. doi:10.1073/pnas.1603142113. 27551098 PMC5018781

[18] Tran C, Hadfield MG. 2011. Larvae of *Pocillopora damicornis* (Anthozoa) settle and metamorphose in response to surface-biofilm bacteria. Mar Ecol Prog Ser 433:85–96. doi:10.3354/meps09192.

[19] Alker AT, Delherbe N, Purdy TN, Moore BS, Shikuma NJ. 2020. Genetic examination of the marine bacterium *Pseudoalteromonas luteoviolacea* and effects of its metamorphosis‐inducing factors. Environ Microbiol 22:4689–4701. doi:10.1111/1462-2920.15211. 32840026 PMC8214333

[20] Guo H, Rischer M, Westermann M, Beemelmanns C. 2021. Two distinct bacterial biofilm components trigger metamorphosis in the colonial hydrozoan Hydractinia echinata. mBio 12:e0040121. doi:10.1128/mBio.00401-21. 34154406 PMC8262903

[21] Freckelton M, Nedved B, Cai Y-S, Cao S, Turano H, Alegado R, Hadfield M. 2019. Bacterial lipopolysaccharide induces settlement and metamorphosis in a marine larva. bioRxiv doi:10.1101/851519. PMC9651628 35467986

[22] Zobell CE, Allen EC. 1935. The significance of marine bacteria in the fouling of submerged surfaces. J Bacteriol 29:239–251. doi:10.1128/jb.29.3.239-251.1935. 16559784 PMC543592

[23] Freckelton M, Nedved BT. 2020. When does symbiosis begin? Bacterial cues necessary for metamorphosis in the marine polychaete *Hydroides elegans*, p 1–15. *In* Cellular dialogues in the holobiont. CRC Press, Boca Raton, FL. doi:10.1201/9780429277375-1.

[24] Aldred N, Nelson A. 2019. Microbiome acquisition during larval settlement of the barnacle *Semibalanus balanoides*. Biol Lett 15:20180763. doi:10.1098/rsbl.2018.0763. 31164063 PMC6597511

[25] Rocchi I, Ericson CF, Malter KE, Zargar S, Eisenstein F, Pilhofer M, Beyhan S, Shikuma NJ. 2019. A bacterial phage tail-like structure kills eukaryotic cells by injecting a nuclease effector. Cell Rep 28:295–301.e4. doi:10.1016/j.celrep.2019.06.019. 31291567

